# Gingivitis Effectiveness of Emulgel Containing 2% Resveratrol in Orthodontic Patients: An 8-Week Randomized Clinical Trial

**DOI:** 10.1155/2021/6615900

**Published:** 2021-03-29

**Authors:** Amin Golshah, Shahla Mirzaeei, Nafiseh Nikkerdar, Fatemeh Ghorbani

**Affiliations:** ^1^Department of Orthodontic, School of Dentistry, Kermanshah University of Medical Sciences, Kermanshah, Iran; ^**2**^ Pharmaceutical Sciences Research Center, Health Institute, Kermanshah University of Medical Sciences, Kermanshah, Iran; ^3^Department of Maxillofacial Radiology, School of Dentistry, Kermanshah University of Medical Sciences, Kermanshah, Iran; ^4^Medical Biology Research Center, Health Technology Institute, Kermanshah University of Medical Sciences, Kermanshah, Iran

## Abstract

**Background:**

Treatment of gingivitis in patients undergoing orthodontic treatment is done through different methods. Anti-inflammatory agents present in toothpaste or mouthwash are applied spontaneously by patients or used as professional treatment protocols. The present study aimed to investigate the gingivitis effectiveness of Emulgel containing 2% resveratrol in orthodontic patients.

**Methods:**

The study was conducted in three groups, namely, experimental, placebo, and control. In the experimental group, participants used an Emulgel containing 2% resveratrol. In the placebo group, subjects used an identical Emulgel without the active components in the test formulations. After brushing their teeth, the participants learned how to use 5 ml of Emulgel on the gums and massage every night for 30 s. In the control group, subjects were instructed to massage their gums for 30 s every night without any product. Evaluated criteria included bleeding on probing (BOP), gingival index (GI), hyperplastic index (HI), and probing pocket depth (PPD).

**Result:**

During 4 and 8 weeks of the study, the PPD score in the control group did not change significantly but decreased in both groups. Also, there was a decrease in the PPD score in the experimental group compared to the placebo and control groups. Similarly, in the experimental group, continuous use of Emulgel containing 2% resveratrol reduced the HI and GI scores significantly at 4 and 8 weeks after the start of the study. Here, the decrease in GI and HI scores in the experimental group was higher than that of the other groups.

**Conclusions:**

The Emulgel containing 2% resveratrol is effective in improving gingival health in orthodontic patients and can lower gingival inflammation over 8 weeks. This trial is registered with the Iranian registry of clinical trials (https://irct.ir/) IRCT20130812014333N91.

## 1. Introduction

The periodontic-orthodontic interrelationship is still a controversial issue despite numerous studies conducted in this regard [1]. Orthodontic treatments may contribute to enhancing periodontal health because they align teeth and balanced occlusion. Therefore, they improve oral hygiene by facilitating access to teeth [2, 3]. However, fixed orthodontic appliances may increase supragingival biofilm accumulation and deteriorate periodontal health [4]. Although it improves dental and skeletal issues, placing the orthodontic appliances in the patient's mouth leads to some changes in oral and gingival hygiene habits [5]. Orthodontic forces represent a physical factor triggering an inflammatory response in the periodontium [6]. The clinical manifestations of gingivitis vary in terms of the severity, distribution, and response to treatment [7]. The effects observed by orthodontics, after the placement of orthodontic appliances can cause inflammatory hyperplasia, alveolar bone loss (periodontitis), and loss of attached gingival support [8–11]. There are several methods to treat gingivitis, including anti-inflammatory agents that are present in toothpaste or mouthwash that are used by patients as professional treatment protocols [11].

Resveratrol (RSV) is a natural multifunctional polyphenol that has therapeutic effects on inflammatory agents at higher concentrations [12, 13]. Resveratrol (3,4,5-trihydroxy-trans-acetylene) was first chemically employed in traditional medicine at the time of its extraction from *Polygonum cuspidatum* (Japanese knot) and *Veratrum grandiflorum* (white rose) [14]. RSV is made by plants as a phytoalexin to react to a stressful stimulus or to microbial or fungal infection. Indeed, plants produce RSV as defense molecules against destructive environmental factors [15, 16]. This natural polyphenol, which has been detected in more than 70 plant species, is also found in discrete amounts in red wines and various human foods [17]. Resveratrol is found in nature as both cis and trans isomers, although the trans isomer is the more abundant and biologically active form [18]. Resveratrol can hinder the growth of bacteria and fungi; reduce virulence factors, biofilm formation, and motility; and affect bacterial susceptibility to various common antibiotics [19]. This compound can be used against various medical situations such as oxidative stress, energy restriction, cancer, and inflammation [20, 21]. Studies have reported the ability of resveratrol to reduce the secretion and expression of inflammatory factors [22]. Resveratrol inhibits the activation of microglia, leading to the release of various proinflammatory factors, the production of reactive oxygen species, and the activation of signal pathways leading to neuroinflammation [23].

To date, there have not been any studies to evaluate the efficiency of Emulgel containing resveratrol in improve clinical gingival inflammatory status. Regarding the anti-inflammatory, antimicrobial, and antifungal properties of resveratrol, this study was conducted for eight weeks to assess the efficiency of a new formulation containing resveratrol in reducing gingival signs of inflammation.

## 2. Methods

### 2.1. Trial Design

This is a parallel, randomized, three-blind, 8-week clinical trial approved by the Ethics Committee of the Kermanshah University of Medical Sciences Research Center. The study was performed on patients with fixed orthodontic appliances with gingivitis in the orthodontic department of Kermanshah Dental School. The participants were asked to sign the informed consent. Afterward, the study was registered at the Iranian Randomized Clinical Trial Center (ID number: IRCT20130812014333N91).

### 2.2. Participants

The participants of this study were selected among healthy subjects between 12 and 25 years old who received fixed orthodontic treatment. Before orthodontic treatment, the subjects had no clinical signs of gingivitis or periodontitis and received oral hygiene instructions by the same clinician. All subjects fulfilled the following criteria for participation in this study: (1) good general health with no history of systemic disease; (2) observing no bone resorptions and periodontitis in the patients according to the radiography (the participants that had periodontitis (loss of clinical attachment >4 mm) and overgrowth of the gums (gingival pocket > 4 mm) being excluded; (3) no periodontal treatment within the last 6 months; and (4) no antibiotic therapy in the last 6 months. Participants underwent orthodontic treatment in the upper and lower arches with monthly follow-up appointments. Clinical evidence of gingivitis was visually screened and was confirmed by the gingival index monthly. Patients with a sign of gingivitis with at least 6 months of orthodontic treatment were selected by convenience sampling. Finally, informed consent was obtained from each patient, followed by explaining the study objectives to them.

### 2.3. Interventions

At the screening visit, all recruited subjects had professional prophylaxis and followed specific oral hygiene instructions. They were then assigned to a base visit (*T*0) for 7–15 days. To control interexaminer variability, BOP, PPD, GI, and HI evaluations were performed by the same examiner (P.M.). At the first visit (*T*0), clinical parameters were measured for all teeth in all subjects. The indices were reevaluated and measured at 4-week (*T*1) and 8-week (*T*2) visits [24].

In the experimental group, subjects used an Emulgel containing 2% resveratrol. In the placebo group, subjects used Emulgel with a formulation similar to the product in the experimental group, but without the active components (without 2% resveratrol). This product was produced by Danesh Bonyan Rahesh Daru Novin Co. in Kermanshah, Iran. Subjects in experimental and placebo groups learned about the 30 s massage of 5 ml of Emulgel on the gums every night after brushing their teeth. Also, subjects in the control group were instructed to massage their gums for 30 s every night without any products. Participants were prohibited from drinking and eating for half an hour.

### 2.4. Study Outcomes

The main objective of this study was to assess the gingivitis effectiveness of an Emulgel containing 2% resveratrol in orthodontic patients. Clinical parameters evaluated were the gingival index (GI), pocket depth exploration (PPD), gingival index (HI), and bleeding in exploration (BOP) during 4 and 8 weeks.

### 2.5. Bleeding on Probing

The absence of BOP can be a predictor of periodontal stability. If the percentage of sites with BOP per person is less than 30% of the total sites explored, it is defined as local bleeding only. About 30% of sites or higher are considered a public BOP [25].

### 2.6. Probing Pocket Depth

PPD (in ml) was recorded at 3 sites around the interdental area (mesiobuccal, midbuccal, and distobuccal sites) [26].

### 2.7. Gingival Index

GI was examined on the facial aspect according to the following:

  Score 0: no inflammation and healthy periodontium
  Score 1: slight edema, a slight change in color, mild inflammation, and no bleeding on probing
  Score 2: moderate change in color and consistency, moderate inflammation, and bleeding on probing
  Score 3: marked redness, severe inflammation, ulceration, spontaneous bleeding, and hypertrophy [27]

### 2.8. Hyperplastic Index

HI was measured visually as follows: (0) no enlargement; (1) gingival enlargement with some distance from the bracket base; (2) enlargement that touches the bracket base or definitely engages the gingival papilla; and (3) enlargement that touches the bracket wings [28].

### 2.9. Sample Size Calculation

The minimum sample size was calculated to be 18 patients based on Abhinav Tadikonda et al. [28]. Here, the standard deviation of GI of the control and test group was assumed to be 0.29 and 0.21, respectively. Also, the mean of GI of the control and test group were considered to be 1.15 and 0.87, respectively, with = 0.1 and alpha = 0.05.

### 2.10. Randomization

Allocation of the bottles to the intervention/placebo/control groups was random with a ratio of 1 : 1:1. After the random sequence generation, a matte bottle was allocated to each patient. We provided the products purposefully in white opaque bottles coded as A, B, or C. Generation of random bottles, their allocation concealment, and implementation were all performed by an independent observer. Patients that selected bottles A, B, and C were considered as the intervention, placebo, and control groups, respectively.

In this study, the allocation was done randomly using Random Allocation Software (version 2.0; Isfahan, Iran) in equal numbers.

### 2.11. Product Features

Prepared Emulgel had a hydrophilic base and was adhesive to the gingival with suitable viscosity, mild orange taste, milky white color, and no allergies. Base ingredients were prepared from a USP-grade excipient. After preparing the formulation, microbial control tests and a one-year stability test were performed. Since there was no similar product in the market, this product was made in Rahesh Daru Novin Company.

### 2.12. Implementation and Blinding

We recorded concomitant medications, medical history, and demographics. Participants, inspectors, and evaluators were blinded to group assignments. The study supervisor assigned the participants to one of the study groups. Each subject was given a unique number that was associated with the assigned product. Participants and researchers were unaware of treatment assignments because of the supervisor's central allocation. The secrecy of the allocation was ensured because the randomization code was not published over the phone until the patient was admitted to the experiment.

### 2.13. The Error of the Method

All orthodontic procedures were performed by one expert orthodontist (A.G.). Measurements and statistical analyses were performed by a student (P.M.). To assess the reliability of the measurements, 50% of the samples (*n* = 35) were measured twice with a 2-week interval.

### 2.14. Statistical Analysis

Statistical analysis was conducted with SPSS (IBM, Armonk, NY). The Kolmogorov–Smirnov test was used to determine whether the variables were normally distributed. If the distribution of variables was normal, the post hoc Tukey's test and one-way analysis of variance (ANOVA) were used to compare the studied groups. A significance level of 0.05 was used for the comparisons.

## 3. Results

### 3.1. Participant Flow

**Figure 1:**
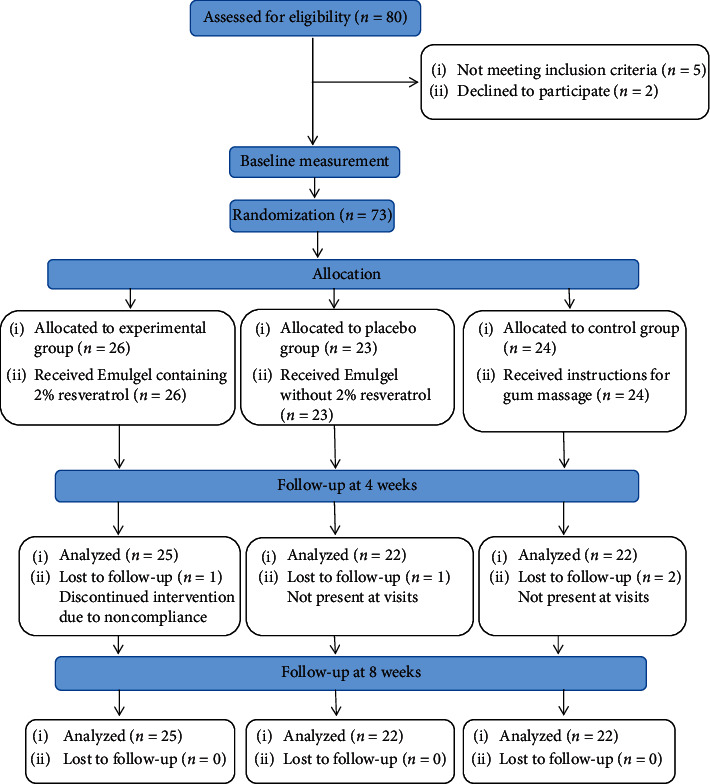
CONSORT flow chart.

Eighty subjects participated in the study, of which 7 were either excluded because they were unwilling to participate or failed to meet the inclusion criteria. In the 4-weeks follow-up, 4 subjects were excluded due to not being present at visits and discontinued intervention due to noncompliance. Finally, 69 participants were assigned to three groups, where 40 (57.9%) were girls and 29 (42.1%) were boys with a mean age of 12–25 years.  Figure 1 presents the flow diagram of the study.

### 3.2. Outcomes

According to the results of the intergroup comparison, in T0, no statistically significant differences were observed between all the evaluated clinical parameters (i.e., PPD, BOP, HI, and GI) in the studied groups (*P* = 0.591).

### 3.3. Probing Pocket Depth

At T1, no statistically significant difference was observed in PPD between the studied groups (*P* = 0.002) such that the mean PPD in the experimental group decreased significantly compared to the control and placebo groups. In T2, no statistically significant difference was observed in PPD between the studied groups (*P* < 0.001). The mean PPD in the experimental group decreased significantly compared to the other two groups. The mean PPD in the placebo group was significantly lower than in the control group.

The intragroup comparison showed that, in the control group, there was no statistically significant difference in PPD during the study. In the placebo and experimental groups, no statistically significant difference was observed in PPD during the study. The mean of this variable in the placebo group decreased significantly in *T*2 compared to *T*0 and *T*1. Also, in the experimental group, the mean of this variable decreased significantly in *T*1 compared to baseline and in *T*2 compared to *T*0 and *T*1 (Table 1).

### 3.4. Hyperplastic Index

The results of the intergroup comparison in T1 indicated no statistically significant difference in HI between the study groups (*P* = 0.442). However, in T2, no statistically significant difference was observed in HI between the study groups (*P* *=* 0.009). In the experimental group, the mean HI was significantly lower than the control and placebo groups.

The intragroup comparison showed that there was no statistically significant difference in HI between the control and placebo groups. Also, in the experimental group, no statistically significant differences were observed in HI so that the mean of this variable decreased in *T*2 compared to *T*0 and *T*1 (Table 2).

### 3.5. Bleeding on Probing

Intergroup comparison showed that, in *T*1, there was no statistically significant difference in BOP between the studied groups (*P* = 0.919) while in *T*2 there was no statistically significant difference in BOP between the studied groups (*P* = 0.806).

The intragroup comparison showed that there was no statistically significant difference in BOP among the studied groups (Table 3).

### 3.6. Gingival Index

Intergroup comparison showed that, in *T*1, there was no significant statistical difference in GI between the studied groups (*P* = 0.173). Also, in *T*2, no statistically significant difference was observed in GI between the studied groups (*P* = 0.010) so that the mean GI in the experimental group was significantly lower than the control group.

The intragroup comparison showed that in the control and placebo groups, there was no statistically significant difference in GI. Moreover, in the experimental group, no statistically significant differences were observed in GI so the mean of this variable decreased in *T*2 compared to *T*0 and *T*1. Meanwhile, the mean of this variable in *T*1 was less than *T*0 (Table 4).

### 3.7. Harms

All the participants were asked if they experience any issues in the usage of the products. . In this respect, none of them complained and reported it was easy and tolerable to use. The use of Emulgel containing 2% resveratrol has no color effect on the gums and teeth and does not cause redness and itching.

## 4. Discussion

In orthodontic treatment, the condition of the gums around the brackets may change due to poor oral hygiene. Oral health in orthodontic patients can be controlled by clinical methods [28]. Abhinav Tadikonda et al. studied the effect of turmeric containing Papain, Bromelain, Miswak, and Neem in limiting plaque and gingivitis. According to their results, this product can be used as a home supplement for clinical treatment in orthodontic patients [28]. The results of studies on chlorhexidine have shown its effectiveness in treating gingivitis. Chlorhexidine-based mouthwash is one of the medical products recommended for the treatment of gingivitis. Chlorhexidine is a synthetic biguanide cationic molecule with strong antibacterial and bacteriostatic action. However, if used for a long time, it can cause dental dyschromia, changes in taste, and plaque deposition [29]. For this reason, over the past decades, researchers have sought alternatives to this molecule. Due to the use of the drug over time, gingivitis can be treated without any restrictions or side effects [29]. In another study, Henrique Pretti et al. investigated the effect of chlorhexidine varnish on gingival growth in orthodontic patients. The results showed that the use of 40% chlorhexidine varnish causes a gradual increase in the clinical crown at 14 and 56 days after use. Chlorhexidine varnish has been proved to be effective against gingival overgrowth in patients undergoing orthodontic treatment [30]. Biocompatibility of dental materials is an important consideration for the patient, clinician, laboratory technician, and manufacturer. Ideally, a dental material that is to be used in the oral cavity should be harmless to all oral tissues, gingiva, mucosa, pulp, and bone [31].

Resveratrol is produced by plants in response to a stressful stimulus or microbial agent and fungal infection to provide plant resistance [32]. Evidence suggests that resveratrol may have a potential therapeutic role in human health because of its anti-inflammatory, antioxidant, antiaging, antidiabetic, anticoagulant, and apoptotic properties [14, 33, 34]. Several clinical trials have been performed on the anti-inflammatory effects of resveratrol. For example, Diego de Sá Coutinho et al. showed that resveratrol can play a role in the treatment of inflammatory processes in chronic diseases and protect cells from inflammation [32]. Moreover, Limagne et al. showed that resveratrol has a strong inhibitory effect on proinflammatory marker secretion and the decrease in IL-6 secretion is dependent on the inhibition of NFkB in the large glands. Such a decrease in IL-6 levels could limit STAT3 activation in macrophages and lead to the rupture of the inflammatory enhancement ring [35]. Overall, these studies show that resveratrol can prevent inflammation and oxidative stress.

This survey assesses the anti-inflammatory effects of 2% resveratrol contamination in orthodontic treatment patients. This was the first clinical study of the effect of emulsifier-containing resveratrol on human periodontitis. Because of the effect of gum massage on the measured indices, a control group was formed to minimize this effect. The results of our study showed that the use of Emulgel containing 2% resveratrol during 8 weeks in patients undergoing orthodontic treatment is effective on PPD, HI, BOP%, and GI such that it successfully reduced the score of these variables. Our study shows that the effectiveness of Emulgel containing 2% resveratrol in reducing some of the symptoms of gingivitis is almost similar to chlorhexidine. Since the use of this Emulgel containing resveratrol is a simple treatment method, its continuous use can mitigate periodontal disease during orthodontic treatment.

### 4.1. Limitations

Our study had several limitations. First, our sample size was small; this study could be done with a larger sample size and compared with compounds such as chlorhexidine. Another limitation of our study was the lack of measuring all factors of gingivitis. Finally, there was a short follow-up.

### 4.2. Generalizability

The generalizability of the results obtained in this study might be limited because this study was performed in one center and by one clinician.

## 5. Conclusions

The results of this clinical trial showed that the Emulgel containing resveratrol is effective in improving gingival health in orthodontic patients for 8 weeks and can reduce gingival inflammation. However, this treatment was not very successful in reducing BOP%.

## Figures and Tables

**Table 1:** Comparison of mean probing pocket depth (PPD) from *T*0 to *T*2 in control, placebo, and experimental groups (mean ± SD).

		*T*_0_		*T*_1_		*T*_2_		*P* value^†^
		Mean	SD	Mean	SD	Mean	SD	
Group	Control	2.28^Aa^	0.29	2.23^Aa^	0.31	2.33^Aa^	0.35	0.342
	Placebo	2.33^Aa^	0.24	2.32^Aa^	0.24	2.20^Ab^	0.20	<0.001
	Experimental	2.23^Aa^	0.43	2.02^Bb^	0.30	1.29^Bc^	0.38	<0.001
*P* value^‡^		0.591		0.002		<0.001		

^†^Repeated measures test followed by Bonferroni's test was used; ^‡^one-way ANOVA test followed by Tukey's test was used. In each row, means with the same lower case superscript letter were not significantly different. In each column, means with the same capital superscript letter were not significantly different.

**Table 2:** Comparison of the mean hyperplastic index (HI) from *T*0 to *T*2 in control, placebo, and experimental groups (mean ± SD).

		*T*_0_		*T*_1_		*T*_2_		*P* value^†^
		Mean	SD	Mean	SD	Mean	SD	
Group	Control	1.50^Aa^	1.10	1.55^Aa^	1.18	1.45^Aa^	0.96	0.940	
	Placebo	1.45^Aa^	1.14	1.41^Aa^	1.18	1.45^Aa^	1.26	0.988	
	Experimental	1.52^Aa^	0.51	1.16^Aa^	0.75	0.68^Bb^	0.63	<0.001	
*P* value^‡^		0.972		0.442		0.009			

^†^Repeated measures test followed by Bonferroni's test was used; ^‡^one-way ANOVA test followed by Tukey's test was used. In each row, means with the same lower case superscript letter were not significantly different. In each column, means with the same capital superscript letter were not significantly different.

**Table 3:** Comparison of mean bleeding on probing (BOP %) from *T*0 to *T*2 in control, placebo, and experimental groups (mean ± SD).

		*T*_0_		*T*_1_		*T*_2_		*P* value^†^
		Mean	SD	Mean	SD	Mean	SD	
Group	Control	15.41^Aa^	8.07	15.65^Aa^	6.86	15.56^Aa^	7.25	0.923
	Placebo	15.09^Aa^	7.05	15.05^Aa^	6.45	14.86^Aa^	6.64	0.875
	Experimental	15.64^Aa^	5.66	14.96^Aa^	5.18	14.36^Aa^	4.87	0.441
*P* value^‡^		0.964		0.919		0.806		

^†^Repeated measures test followed by Bonferroni's test was used; ^‡^one-way ANOVA test followed by Tukey's test was used. In each row, means with the same lower case superscript letter were not significantly different. In each column, means with the same capital superscript letter were not significantly different.

**Table 4:** Comparison of the mean gingival index (GI) from *T*0 to *T*2 in control, placebo, and experimental groups (mean ± SD).

		*T*_0_		*T*_1_		*T*_2_		*P* value^†^
		Mean	SD	Mean	SD	Mean	SD	
Group	Control	1.01^Aa^	0.41	1.06^Aa^	0.39	1.03^Aa^	0.36	0.621
	Placebo	1.03^Aa^	0.41	0.99^Aa^	0.36	0.98^ABa^	0.37	0.709
	Experimental	1.00^Aa^	0.32	0.88^Ab^	0.21	0.77^Bc^	0.15	<0.001
*P* value^‡^		0.966		0.173		0.010		

^†^Repeated measures test followed by Bonferroni's test was used; ^‡^one-way ANOVA test followed by Tukey's test was used. In each row, means with the same lower case superscript letter were not significantly different. In each column, means with the same capital superscript letter were not significantly different.

## Data Availability

No data were used to support this study.
